# Genome-Wide Analysis of the *PYL* Gene Family and Identification of *PYL* Genes That Respond to Abiotic Stress in *Brassica napus*

**DOI:** 10.3390/genes9030156

**Published:** 2018-03-12

**Authors:** Feifei Di, Hongju Jian, Tengyue Wang, Xueping Chen, Yiran Ding, Hai Du, Kun Lu, Jiana Li, Liezhao Liu

**Affiliations:** 1 College of Agronomy and Biotechnology, Chongqing Engineering Research Center for Rapeseed, Southwest University, Chongqing 400716, China; sddifeifei@163.com (F.D.); jianhongju1989@126.com (H.J.); tengyue1992@126.com (T.W.); xpchen19925569@163.com (X.C.); rapran@163.com (Y.D.); haidu81@aliyun.com (H.D.); drlukun@swu.edu.cn (K.L.); ljn1950@swu.edu.cn (J.L.); 2 Academy of Agricultural Sciences, Southwest University, Chongqing 400716, China

**Keywords:** *Brassica napus*, abscisic acid, *PYL*, gene expression pattern, abiotic stress

## Abstract

Abscisic acid (ABA) is an endogenous phytohormone that plays important roles in the regulation of plant growth, development, and stress responses. The pyrabactin resistance 1-like (PYR/PYL) protein is a core regulatory component of ABA signaling networks in plants. However, no details regarding this family in *Brassica napus* are available. Here, 46 *PYLs* were identified in the *B. napus* genome. Based on phylogenetic analysis, *BnPYR1* and *BnPYL1-3* belong to subfamily I, *BnPYL7-10* belong to subfamily II, and *BnPYL4-6* and *BnPYL11-13* belong to subfamily III. Analysis of *BnPYL* conserved motifs showed that every subfamily contained four common motifs. By predicting cis-elements in the promoters, we found that all *BnPYL* members contained hormone- and stress-related elements and that expression levels of most *BnPYLs* were relatively higher in seeds at the germination stage than those in other organs or at other developmental stages. Gene Ontology (GO) enrichment showed that *BnPYL* genes mainly participate in responses to stimuli. To identify crucial *PYLs* mediating the response to abiotic stress in *B. napus*, expression changes in 14 *BnPYL* genes were determined by quantitative real-time RT-PCR after drought, heat, and salinity treatments, and identified *BnPYR1-3*, *BnPYL1-2*, and *BnPYL7-2* in respond to abiotic stresses. The findings of this study lay a foundation for further investigations of *PYL* genes in *B. napus*.

## 1. Introduction

Abscisic acid (ABA) is an important plant hormone that plays roles not only in plant growth and development processes, such as cell division and elongation, stomatal movement, seed dormancy, embryo development, and old leaf abscission [1,2,3,4], but also in response to biotic and abiotic stresses [5]. ABA is sensed by the ABA receptor PYR/PYL (pyrabactin resistance 1-like) family in the ABA core signal transduction pathway [6,7,8]. When bound by ABA, PYR/PYL inhibits the enzymatic activity of protein phosphatase 2C (PP2C), leading to the release of the serine/threonine-protein kinase SRK2 (SnRK2) [6]. SnRK2 kinases are activated via activation loop autophosphorylation [9], and activated SnRK2 kinases subsequently phosphorylate transcription factors, such as the ABA-responsive element binding factor (ABF), which is thought to be necessary to activate ABFs [10,11]. These activated ABFs enter the nucleus to up-regulate the expression of downstream ABA-induced stress-associated genes.

Plants increase intracellular ABA content via ABA biosynthesis when subjected to abiotic stresses, such as drought, high and low temperatures, salinity, and heavy metals. Large quantities of synthetic ABA bind to PYLs, which perform ABA signal transduction and respond to stress [12]. The initial step and motivation for ABA signal transduction is ABA binding to PYLs; therefore, PYLs play important roles in this signal transduction pathway. Fourteen *PYLs* with highly conserved amino acid sequences have been identified in *Arabidopsis thaliana* and named *PYR1* and *PYL1-13* [6,8,13]. Furthermore, orthologous genes in other crops have been reported, including six *PYLs* in sweet orange [14], eight *PYLs* in grape [15], 21 *PYL* homologs in soybean [16], 12 *PYLs* in rice [17], 14 *PYLs* in tomato [18], 14 *PYLs* in rubber tree [19], 24 *PYLs* in *Brassica rapa* [20], and 27 *PYLs* in cotton [21]. These genes have been categorized into three subfamilies, and the functions of some *PYL* genes in plants have been characterized successfully. Overexpression of *AtPYL4* in *A. thaliana* enhances its drought tolerance [22]. The drought and salt stress tolerance of *Oryza sativa* was enhanced by overexpressing *OsPYL5* [23]. *PYL9* promotes drought resistance, and *PYL8*, together with *PYL9*, plays a vital role in regulating lateral root growth in *A. thaliana* [24,25]. These results all suggest that *PYL* genes play a role in enhancing tolerances under abiotic stress. The identification and characterization of *PYLs* in these plants thus plays a very important role in understanding their function and the ABA signal transduction pathway. However, little information is available about *PYLs* in *Brassica napus*.

*B. napus* L. (AACC, 2*n* = 38), which belongs to Brassicaceae, is an allotetraploid species that are formed by an interspecific natural cross between *B. rapa* (AA, 2*n* = 20) and *Brassica oleracea* (CC, 2*n* = 18) and subsequent chromosome doubling approximately 7500 years ago [26]. Rapeseed is the third largest source of vegetable oil globally, after palm and soybean (http://faostat3.fao.org). *B. napus* provides not only high-quality oil with low levels of saturated fatty acids and cholesterol and high microelement content, but also meals for animal feed and a source of biodiesel. *B. napus* plants often suffer from various biotic and abiotic stresses due to environmental changes, affecting oilseed yield. Identifying *PYLs* in *B. napus* not only lays a foundation for understanding ABA signaling, but also provides information for defending against stresses.

In this study, we identified *PYL* genes in *B. napus* by protein basic local alignment search tool (BLASTP) searches of the recently completed *B. napus* genome [26] using the 14 PYL protein sequences from *A. thaliana* as queries. We analyzed phylogenetic trees, exon-intron structures, conserved protein motifs, promoter elements, and gene expression profiles in various tissues and organs, as well as Gene Ontology (GO) and micro RNA (miRNA) targeting of the *BnPYL* genes to further characterize *BnPYLs*. In addition, we analyzed the gene expression patterns of some *BnPYLs* under different abiotic stresses, including heat, drought, and salinity treatments. Our study provides insights into the *PYL* gene family in *B. napus*.

## 2. Materials and Methods

### 2.1. Plant Materials and Stress Treatments

Healthy *B. napus* ZS11 seeds were germinated on petri dishes soaked in water for 48 h and sown in 10 cm plastic pots. Seedlings were grown to the four-leaf-stage in a chamber room (16 h day/8 h dark at temperature 25 °C) and then treated with various stresses. The seedlings were irrigated with 20% polyethylene glycol-6000 (PEG-6000) or 200 mM NaCl for drought and salinity abiotic stress, respectively. Seedlings were subjected to 40 °C for high-temperature stress, and leaf samples were collected at 3, 6, and 12 h. Young leaves were collected at 3, 6, 12, 24, 48, and 72 h after drought treatment. Salinity-treated leaves were collected at 3, 6, 12, 24, and 48 h. The collected leaves were immediately frozen in liquid nitrogen and stored in a −80 °C freezer for RNA isolation.

### 2.2. Genome-Wide Identification and Chromosomal Location of PYL Gene Family in B. napus

To better understand the *BnPYL* gene family, we used 14 PYL protein sequences from the *A. thaliana* genome (http://www.arabidopsis.org/) as queries to identify *PYL* genes in *B. napus*, *B. rapa* and *B. oleracea* via protein basic local alignment search tool (BLASTP) [27]. The top *E*-value was less than 1 × 10^−20^. Some redundant genes were removed manually because of the complexity of the allotetraploid *B. napus* genome. The related gene sequences and positions were identified in BRAD (http://brassicadb.org/) and the *B. napus* Genome Browser (http://www.genoscope.cns.fr/brassicanapus/). The number of amino acids in a sequence and its isoelectric point (pI) and molecular weight (MW) were searched at the ExPASy website (http://web.expasy.org/). The chromosomal distributions of 46 *BnPYLs* were drawn using MapChart software based on their chromosomal position [28].

### 2.3. Phylogenetic Tree Analysis of PYL Gene Family in B. napus, B. rapa, B. oleracea and A. thaliana

To understand the evolutionary relationships of the *PYL* gene family, we used *B. napus*, *B. rapa*, *B. oleracea*, and *A. thaliana* protein sequences to build a phylogenetic tree. The protein sequences were multiple-aligned using MEGA 5.2 software [29]. The phylogenetic tree was built based on the neighbor-joining (NJ) method with 1000 bootstrap replicates. We then uploaded the tree diagram file (*.nwk) from MEGA to the iTOL website (http://itol.embl.de/) to better visualize the phylogenetic tree.

### 2.4. Analysis of Gene Exon-Intron Structures and Protein Conserved Motifs

Gene exon-intron structures were analyzed using the Gene Structure Display Server (GSDS2.0) [30] by comparing the codon sequences and genomic sequences of the 46 *BnPYL* members. Related gene sequences were searched on the *B. napus* Genome Browser (http://www.genoscope.cns.fr/brassicanapus/). Motifs were identified in Multiple EM for Motif Elicitation version 4.11.4 (MEME) [31] by analyzing 46 full-length *BnPYL* protein sequences. A limit of twenty motifs was set, and any number of repetitions was expected as motif sites were distributed throughout the sequences.

### 2.5. Analyzing cis-Elements in the BnPYL Promoters

We analyzed the cis-elements of *BnPYL* promoters to further understand the *BnPYL* gene family. We examined the sequences within 1500 base pairs (bp) upstream of initiation codons (ATG) for promoter analysis and were searched for these sequences in the *B. napus* Genome Browser. The cis-elements in promoters were subsequently searched using the PlantCARE database (http://bioinformatics.psb.ugent.be/webtools/plantcare/html/).

### 2.6. Prediction of miRNAs Targeting BnPYL Genes

In this study, all of the genome sequences of *BnPYL* family genes were submitted as candidate genes for predicting potential miRNAs by searching against the available *B. napus* reference of miRNA sequences using psRNATarget Server with default parameters [32]. Cytoscape software [33] was used to visualize the interactions between the predicted miRNAs and the corresponding target *BnPYL* genes.

### 2.7. Analysis of Gene Expression Profiles and Gene Ontology Enrichment

To further characterize the different temporal and spatial gene expression patterns of the *BnPYL* gene family, we analyzed RNA sequencing (RNA-seq) data. Transcriptome sequencing datasets were deposited in the BioProject ID PRJNA358784, which was used to perform RNA-seq of different *B. napus* cultivar ZS11 tissues. We analyzed the total RNA-seq data of the roots, stems, leaves, flowers, seeds, and siliques at the germination, seedling, bud, initial flowering, and full-bloom stages. We quantified these gene expression levels on the basis of their fragments per kilobase of exon per million reads mapped (FPKM) values using Cufflinks with default parameters [34], and represented these results using HemI 1.0 software [35]. To further understand the functions of these genes, we used BLAST software (https://blast.ncbi.nlm.nih.gov/) to align the *BnPYL* sequences with entries in the NCBI nonredundant (NR) protein [36], Swiss-Prot [1], GO [37], clusters of orthologous groups (COG) [38], eukaryotic orthologous groups (KOG) [39], Protein family (Pfam) [40], and Kyoto encyclopedia of genes and genomes (KEGG) databases [41], and conducted GO enrichment analysis of those *BnPYLs* that were annotated. GO enrichment was performed using the BinGO program of Cytoscape_3.4.0 software [33] with an FDR < 0.01.

### 2.8. RNA Extraction and Real-Time RT-PCR

RNA was extracted from drought-, heat- and salinity-treated samples using an EZ-10 DNAaway RNA Mini-prep Kit (Sangon Biotech, Shanghai, China), according to the manufacturer’s instructions. RNA concentrations were measured by a NanoDrop 2000 (Thermo Fisher Scientific, Worcester, MA, USA), and RNA integrity was evaluated by electrophoresis. One microgram of RNA template from each sample was used to synthesize the first-strand of complementary DNA (cDNA) using an iScript^TM^ cDNA Synthesis Kit (Bio-Rad, Hercules, CA, USA), and the cDNA solution was then diluted 20 times with distilled deionized water. Each reaction had a final volume of 20 µL and contained 2 µL of 20-fold-diluted cDNA solution, 10 µL of SYBR^®^ Green Supermix (Bio-Rad), 0.4 µL of 10 mM forward and reverse primers, and 7.2 µL of distilled deionized water. We performed qRT-PCR on a CFX96 Real-time System (Bio-Rad), according to the manufacturer’s instructions. The qRT-PCR program was as follows: 98 °C for 30 s, then 40 cycles of 98 °C for 15 s, 60 °C for 30 s, and an increase from 65 °C to 95 °C with an increment of 0.5 °C every 0.05 s. Three technical replications were performed per sample. We calculated the relative gene expression levels based on the 2^−ΔΔCt^ method using *Actin7* of *B. napus* as an internal control for normalizing gene expression levels. RT-PCR primers were designed on Primer Premier Software (version 5.0). Given the highly homologous candidates in *B. napus*, all of the primer sequences avoided false priming and are listed in Table S1. All of the qRT-PCR results were displayed using HemI 1.0 software [35].

## 3. Results

### 3.1. Characterization of BnPYL Gene Family

**Figure 1:**
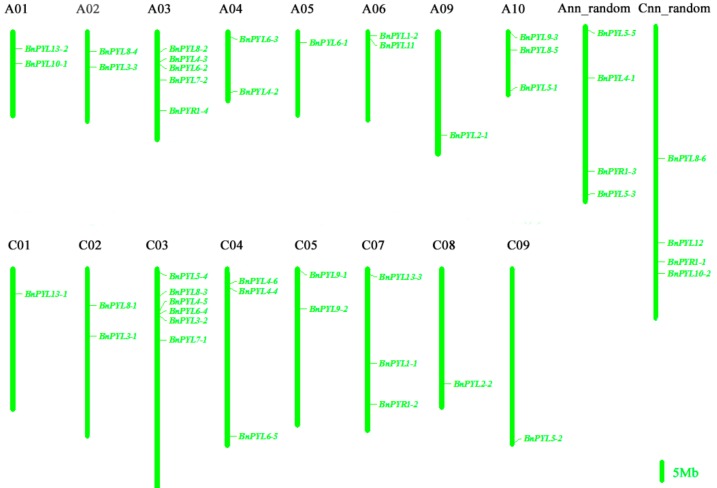
*BnPYL* distributions on *B. napus* chromosomes. The chromosome name is at the top of each bar. Ann_random: unmapped A chromosomes of the *B. napus* genome; Cnn_random: unmapped C chromosomes of the *B. napus* genome; the scale of the chromosome is in millions of bases (Mb).

In this study, we identified 46 *PYL* genes in the *B. napus* genome through BLASTP by using 14 *AtPYL* protein sequences as references (Table 1). Every member of the 14 *AtPYLs* was homologous to one to six sequences in *B. napus* genome. For example, *AtPYL4*, *AtPYL6* and *AtPYL8* had six homologs in *B. napus*, but *AtPYL11* and *AtPYL12* had only one homolog. We found that 46 *BnPYL* members were all derived from a progenitor by comparing the composition of these *PYL* genes in *B. napus* and their relatives in its two ancestors *B. rapa* and *B. oleracea*. The *B. rapa* genome contains 22 *BnPYLs* and the *B. oleracea* genome contains 24 *BnPYLs*. Based on the physical positions of the 46 *BnPYL* genes, 38 *BnPYLs* were accurately mapped onto the 19 *B. napus* chromosomes, whereas the remaining *BnPYLs* were located on the unmapped scaffolds in the Ann_random and Cnn_ random genomes (Figure 1). *BnPYLs* are distributed on all *B. napus* chromosomes, except A07, A08, and C06, and were densely distributed on A03 and C03, containing five and six members, respectively (Figure 1). Table 1 shows that the gene lengths range from 489 (*BnPYL11*) to 2229 (*BnPYL8-3*), with one to four exons in each sequence. The transcripts, except for introns and noncoding regions (UTR), consist of coding DNA sequences (CDS) with sizes varying from 489 (*BnPYL11*) to 648 bp (*BnPYL1-1*). The lengths of the corresponding *BnPYL* protein sequences range from 162 (*BnPYL11*) to 215 (*BnPYL1-1*) amino acids. The average MW was 21.64 kDa. The pI values of these proteins range from 5.12 (*BnPYL13-1*) to 8.91 (*BnPYL3-3*), and 89.13% of these proteins are acidic (pI < 7).

### 3.2. Analysis of Phylogenetic Relationships and Gene Structures of BnPYLs

**Figure 2:**
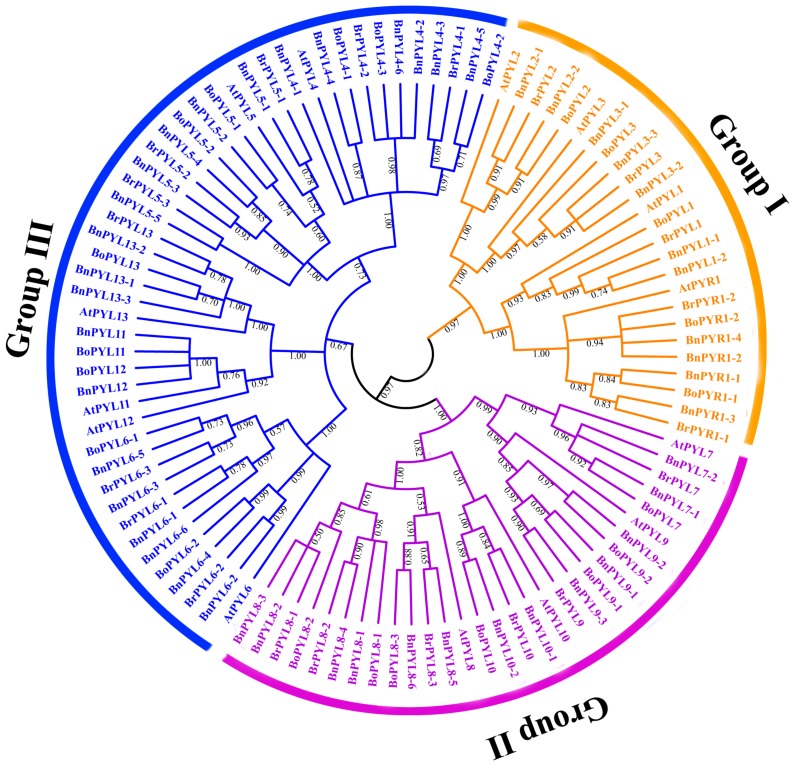
Phylogenetic tree analysis of *PYLs* in *Arabidopsis thaliana*, *B. napus*, *Brassica rapa* and *Brassica oleracea*. In total, 14 *AtPYLs* from *A. thaliana*, 24 *BrPYLs* from *B. rapa*, 23 *BoPYLs* from *B. oleracea* and 46 *BnPYLs* from *B. napus* were included. These 107 sequences were used to construct a neighbor-joining (NJ) tree. The tree was divided into three groups, represented by different colors.

**Figure 3:**
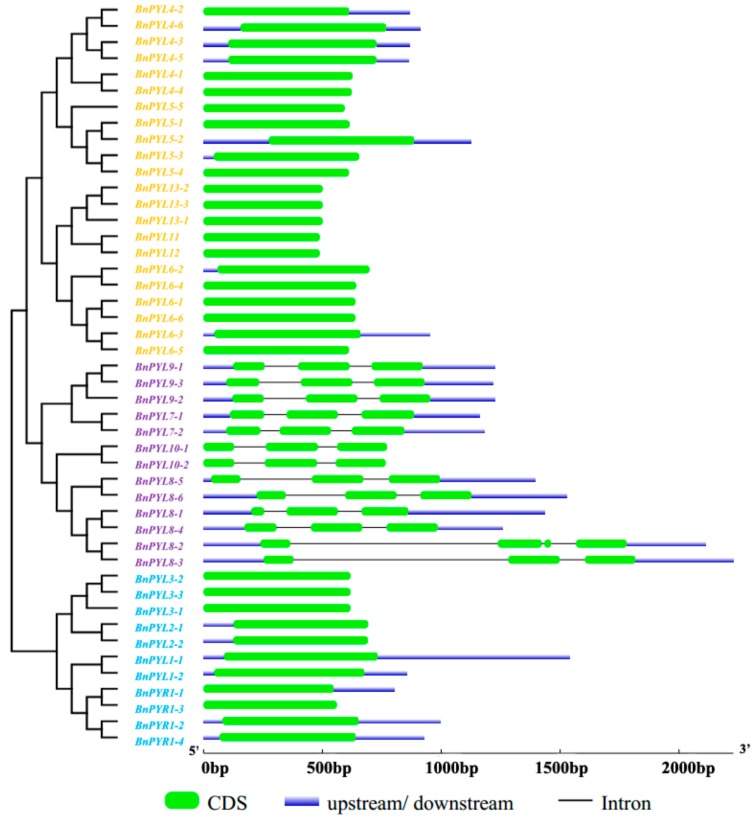
The exon-intron structure of *BnPYLs* according to their phylogenetic relationships. Light yellow *BnPYLs* represent Group III; *BnPYLs* in light purple belong to Group II; and *BnPYLs* in light blue are Group I. The lengths and positions of introns and exons are shown on the figure. The green boxes and gray lines denote exons and introns, respectively. CDS: coding sequences; bp: base pairs.

To study the evolutionary relationships between *BnPYLs* and *PYLs* from *A. thaliana*, *B. rapa* and *B. oleracea*, the 46 *BnPYLs* with 14 *AtPYL*, 22 *BoPYL*, and 20 *BrPYL* were clustered into three groups, designated Group I, Group II, and Group III, on an unrooted phylogenetic tree, with at least 67% bootstrap support for Group III. *PYR1* and *PYL1-3* belong to Group I, *PYL7-10* belong to Group II, and *PYL4-6* and *PYL11-13* belong to Group III (Figure 2). Overall, Group I contained 25 members, Group II contained 30 *PYLs*, and Group III comprised 47 *PYLs*. Specifically, 11, 13, and 22 *BnPYLs* were found to be distributed into Groups I, II, and III, respectively. *PYLs* grouping into the same subfamilies may have similar functions. To understand the *PYL* gene structures, we analyzed the *BnPYL* gene exon-introns using the GSDS website. We presented these structural features based on evolutionary tree relationships. In Figure 3, Groups I and III do not possess introns, and all of the Group II members, except *BnPYL8-2*, have two introns each. *BnPYL8-2* contains three introns (Figure 3). These results indicated that members within a single subfamily had highly similar gene structures, which is consistent with their phylogenetic relationships.

### 3.3. Analysis of BnPYL Conserved Motifs

**Figure 4:**
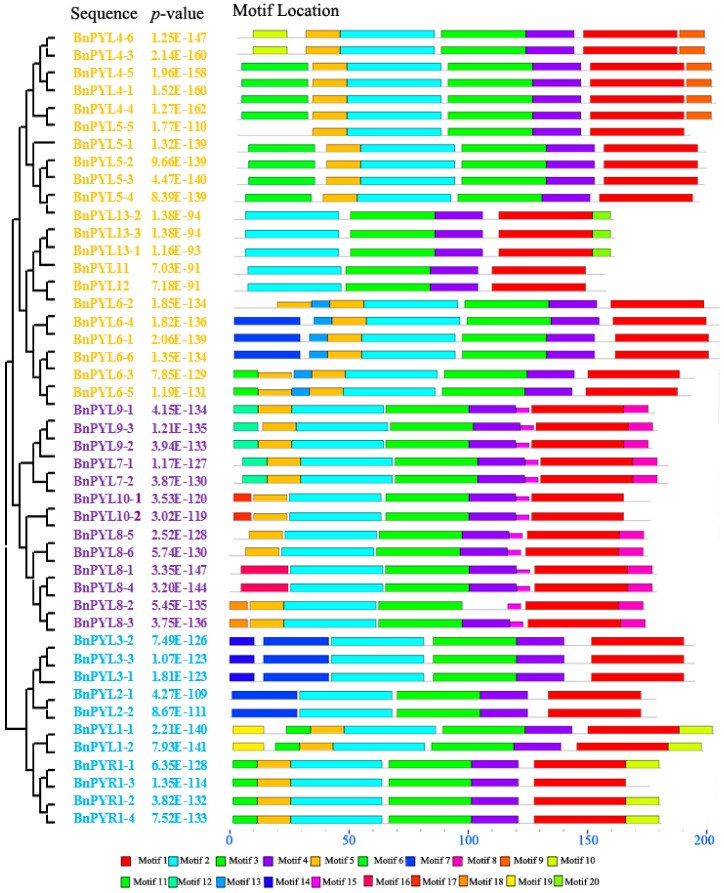
The conserved motifs of the BnPYL proteins presented according to their phylogenetic relationships. These motifs were identified using Multiple EM for Motif Elicitation (MEME), and boxes of different colors represent different motifs.

We analyzed full-length protein sequences of 46 BnPYLs using MEME software to identify their conserved motifs. Twenty conserved motifs were recognized, and the length of the motifs range from 8 to 41 amino acids. Every BnPYL member contains from four to eight conserved motifs (Figure 4). Motifs 1, 2, and 3 are present in all 46 BnPYL proteins, and three motifs contained a START-like domain. All the proteins except BnPYL8-2 show motif 4. Furthermore, all the members of Group II contain motif 8. We found that every subfamily possesses four identical motifs, suggesting that PYL proteins have highly conserved amino acid residues and members of the same group may have similar functions. In addition, we compared the three motifs that are common to all BnPYL proteins with sequences from another study and found that those amino acids marked with asterisks in Figure S1 are similar to the sequences in the previous study [20], suggesting that these residues may be play a role in receptor activation.

### 3.4. Cis-Elements in BnPYL Promoters

To better understand the transcriptional regulation and potential function of the *BnPYL* genes, we isolated sequences within 1500 bp upstream of the initiation codons of *BnPYLs* and identified cis-elements within these promoter sequences using the PlantCARE database. We analyzed ten hormone-related and five stress-related elements (Table S2). The upstream regions of all *BnPYL* members contain at least two hormone-related elements, such as abscisic acid-responsive (ABRE), auxin-responsive (AUXRR-core, TGA-element), MeJA-responsive (CGTCA-motif, TGACG-motif), ethylene-responsive (ERE), gibberellin-responsive (GARE, P-box, TATC-box), and salicylic acid-responsive elements (TCA-element). In addition, the 46 *BnPYL* promoters contain one or more stress-related elements, such as fungal elicitor-responsive (Box-W1/W3), heat stress-responsive (HSE), and low-temperature-responsive (LTR) elements and a MYB-binding site that is involved in drought-inducibility (MBS), defense, and stress responsiveness (TC-rich repeats). The four most abundant hormone-related elements in the 46 *BnPYLs* are ABRE, CGTCA-motif, TGACG-motif, and TCA-element, and the three most abundant stress-related elements are HSE, MBS, and TC-rich repeats. The MBS element was found in 38 of 46 *BnPYLs*, suggesting that *BnPYLs* may play an important role in regulating drought stress. *BnPYL8-3* contains ten MeJA-related elements, suggesting that *BnPYL8-3* may respond to MeJA exposure. Similarly, *BnPYL8-5* contains seven MBS elements and may be related to drought tolerance. The diversities of the hormone- and stress-related cis-elements in the *BnPYL* promoters suggested that expression may differ in response to hormones and stresses.

### 3.5. Comprehensive Analysis of microRNA Targeting BnPYL Genes

**Figure 5:**
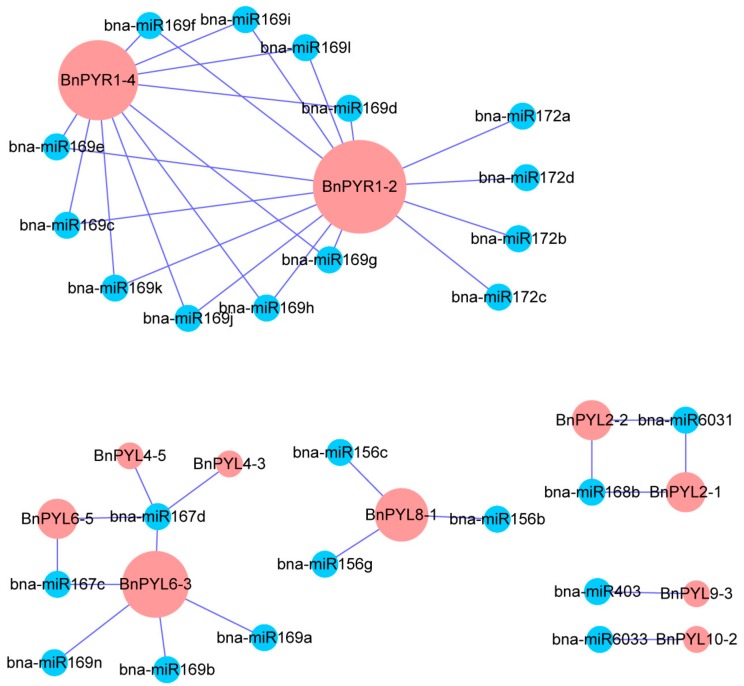
A schematic representation of the regulatory network relationships between the putative miRNAs and their targeted *BnPYL* genes.

In recent years, a considerable number of studies have shown that miRNAs mainly respond to stress by regulating the expression of genes associated with stress in plants. To understand the underlying regulatory mechanism of miRNAs involved in the regulation of *BnPYLs*, we identified 26 putative miRNAs targeting 11 *BnPYL* genes to construct a relationship network using Cytoscape software (Figure 5). We analyzed the connection distribution of the regulation network and found *BnPYR1-2* and *BnPYR1-4* are the most targeted *BnPYL* genes for successful targeting by *B. napus* miRNAs. Ten members of the miRNA169 family and four members of the miRNA172 family target *BnPYR1-2*, and 10 members of the miRNA169 family target *BnPYR1-4*. Notably, miR169 plays important roles in *A. thaliana* by targeting genes related to drought stress [42]. In addition, *BnPYL2-1*, *BnPYL2-2*, *BnPYL4-3*, *BnPYL4-5*, *BnPYL6-3*, *BnPYL6-5*, *BnPYL8-1*, *BnPYL9-3*, and *BnPYL10-2* were regulated by different miRNAs. Furthermore, miR167d was identified as an miRNA targeting four *BnPYL* genes (*BnPYL4-3*, *BnPYL4-5*, *BnPYL6-3*, and *BnPYL6-5*), the most targeted *BnPYL* genes in our study.

### 3.6. Analysis of BnPYL Expression Levels in Tissues

**Figure 6:**
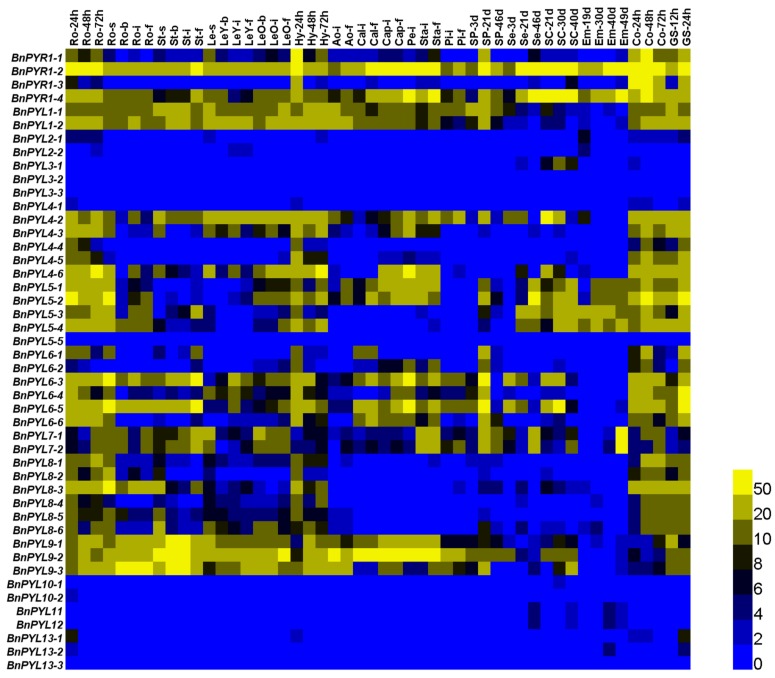
Expression levels of *BnPYL* genes in different tissues and at different stages of *B. napus.* Ro, root; St, stem; Le, leaf; Hy, hypocotyl; Ao, anthocaulus; Cal, calyx; Cap, capillament; Pe, petal; Sta, stamen; Pi, pistil; SP, silique; Se, seed; SC, seed coat; Em, embryo; Co., cotyledon; GS, germinate seed. s, seedling stage; b, bud stage; i, initial flowering stage; and, f, full-bloom stage. The 24, 48, and 72 h labels indicate the time that had passed after seed germination. The 3, 19, 21, 30, 40, and 46 d labels indicate the number of days that had passed after the flowering stage. The bar on the lower right corner represents fragments per kilobase of exon per million reads mapped (FPKM) values, and different colors represent different expression levels.

To characterize the expression of the *BnPYL* gene family, we analyzed 50 different tissues and organs of *B. napus* at different development stages based on RNA-seq datasets from *B. napus* ZS11 (BioProject ID PRJNA358784); the reliability of the datasets was verified by Zhou et al. using qRT-PCR [34]. The expression levels of most *PYL* members differed in the different tissues and organs, suggesting that different functions were required in different tissues. Notably, the expression levels of most *PYL* genes in seeds (roots, hypocotyl, cotyledon and germinated seed) at the germination stage were higher than those of other organs and of plants at other developmental stages (Figure 6 and Table S3). Thirteen *BnPYL* genes (*PYR1-2*, *PYR1-4*, *PYL1*, *PYL4-2*, *PYL4-6*, *PYL5-2*, *PYL5-4*, *PYL6-3*, *PYL6-5*, and *PYL9*) were highly expressed in nearly all tissues, suggesting that these genes play an important role in regulating *B. napus* biology process. By contrast, the expression abundances of 14 *PYLs* (*PYL2*, *PYL3*, *PYL4-1*, *PYL5-5*, *PYL10*, *PYL11*, *PYL12*, and *PYL13*) in all tissues were very low, with nearly no expression in *B. napus*. The expression levels of the same gene in the same tissues or organs differed in different growth stages, suggesting that some genes are expressed at specific times.

### 3.7. Gene Ontology Enrichment

To further understand the functions of the *BnPYLs*, we performed GO annotation and GO enrichment analyses. The GO terms included three categories, biological process (BP), molecular function (MF), and cellular component (CC). GO enrichment confirmed that these 46 *BnPYLs* are enriched in the cell (GO:0005623), cell part (GO:0044464) and organelle (GO:0043226) terms of the CC category. MF is enriched in binding (GO:0005488). Biological regulation (GO:0065007) and response to stimulus (GO:0050896) were the most abundant functions in the BP category (Table S4). The GO enrichment suggested that *BnPYLs* play important roles in responding to stress, consistent with the findings of a previous study [5].

### 3.8. The Expression Patterns of PYLs in B. napus under Abiotic Stress

**Figure 7:**
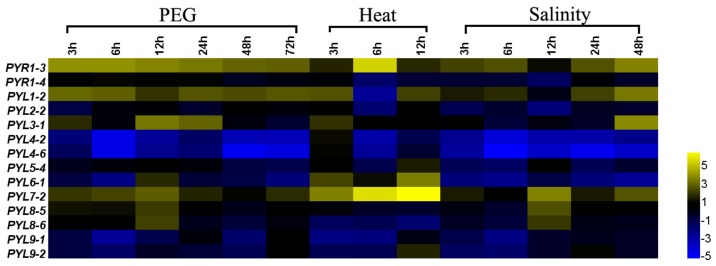
*PYL* expression levels under drought, salinity and heat abiotic stresses in *B. napus*. The bars display the relative gene expression levels, calculated based on the 2^−ΔΔCt^ method. The expression level is equal to the mean values and transform log_2_ values. polyethylene glycol (PEG), drought stress; Heat, high-temperature stress; Salinity, salt stress.

To further explore *BnPYL* gene expression patterns under abiotic stresses and identify genes important for improving tolerance to abiotic stresses, *B. napus* seedlings were subjected to abiotic stresses such as drought, salinity, and heat. A total of 14 *BnPYLs* were selected to perform quantitative real-time RT-PCR at different time points after various abiotic treatments, and the expression levels of these genes are listed in Table S5. The expression patterns of selected 14 *BnPYL* genes showed transcriptional changed under drought, heat, and salinity stresses, and this suggested that the response of *BnPYLs* to multiple stresses is a dynamic process (Figure 7). For drought treatment, three genes (*PYR1-3*, *PYL1-2* and *PYL7-2*) of the 14 selected genes had similar expression patterns; specifically, they tended to be up-regulated at all of the time points. The expression levels of *PYL3-1*, *PYL6-1*, *PYL8-5*, and *PYL8-6* increased at one or three time points. The seven up-regulated genes (*PYR1-3*, *PYL1-2*, *PYL3-1*, *PYL6-1*, *PYL7-2*, *PYL8-5*, and *PYL8-6*) were highly induced in response to drought treatment at 12 h than at other time points. The change of *PYR1-4* expression levels is not significantly at all time points under drought stress. By contrast, the expression levels of other six *BnPYL* genes (*PYL2-2*, *PYL4-2*, *PYL4-6*, *PYL5-4*, *PYL9-1*, *PYL9-2*) were generally down-regulated under drought treatment. For the up-regulated *BnPYL* genes after drought treatment, they may be related to drought tolerance. Under heat stress, 14 *BnPYL* genes showed different expression levels (Figure 7). *PYR1-3*, *PYL1-2*, *PYL3-1*, *PYL4-2*, *PYL5-4*, *PYL6-1*, *PYL7-2*, and *PYL 9-2* were up-regulated, and the expression levels of *PYR1-3* at 6 h and *PYL7-2* at three-time points considerably up-regulated under heat treatment. The other genes were down-regulated after heat treatment. For salinity stress, *PYR1-3*, *PYL1-2*, *PYL3-1*, *PYL7-2*, *PYL8-5*, and *PYL8-6* were up-regulated at specific time points or during periods of time, while the other genes were down-regulated. The expression level of *PYL3-1* was stable or down-regulated in the 24 h after treatment, but it was up-regulated at 48 h, which suggested that some *PYLs* may be induced under serious stress conditions. The expression levels of *PYR1-3* and *PYL1-2* increased during the early stages, stabilized at 12 h and increased at 24 and 48 h, which showed the complexity of the regulatory networks in responding to stresses. Taken together, we found the expression patterns of some *BnPYL* genes are similar under drought, salinity, and heat treatments (Figure 7). In addition, our results suggested that the expression levels of *PYR1-3*, *PYL1-2*, and *PYL7-2* were induced by drought, high-temperature, and salinity stresses, suggesting that these genes might be important candidates for improving tolerance to abiotic stresses.

## 4. Discussion

The phytohormone ABA is well known for its two functions: the regulation of plant growth and development and the responding to abiotic and biotic stresses. In our research program, we had a wilting mutant of *B. napus* through Ethyl methanesulfonate (EMS) mutagenesis. The wilting mutant accumulated a higher content of ABA than the wild type, and the expression of *PYL* genes were up-regulated compared with wild type. PYL as ABA receptor is the first step for the downstream ABA signaling, and an important element in the core ABA signal transduction pathway. Although *PYLs* have been identified in many plants, this work is the first identification of *PYL* genes in *B. napus*.

### 4.1. Characterization of PYL Gene Family in Brassica napus

*B. napus* as an allotetraploid species that experienced widespread genome duplication and merging events [26]. According to our results, the number of *PYL* genes in *B. napus* far exceeds the 14 *AtPYLs* in *A. thaliana* [8], suggesting that genome duplication may have occurred in the evolution of *B. napus*. Each *PYL* in *A. thaliana* was typically homologous to 2-6 genes in the *B. napus* genome, consistent with the finding that one *A. thaliana* gene corresponded to two or more homologous genes in *B. napus* [43]. Based on phylogenetic analysis, the 46 *BnPYLs* were classified into the same three groups as *PYL* genes from *A. thaliana* (Figure 2), suggesting similar evolutionary trajectories between *B. napus* and *A. thaliana*. In addition, the results indicated that the encoding *BnPYL* genes, which are homologous to *A. thaliana* genes, might play similar roles in specific biological processes. These groupings in the phylogenetic tree were supported by the exon-intron structure (Figure 3). These clusters of subfamilies were consistent with the groupings of *B. rapa* and tomato [18,20], but were different from the groupings of the rubber tree genes, which were divided into the groups *HbPYL1-3*, *HbPYL4-7*, and *HbPYL8-14* [19]. This difference may be caused by the sequence diversity of the various species. In addition, the numbers and composition of motifs were varied in each *BnPYL* family. Motif 1, motif 2, and motif 3 with 41, 41, and 37 amino acid residues, respectively (Figure S1) were detected in all BnPYL protein sequences (Figure 4), indicating that BnPYLs have a highly conserved protein structures. Phylogeny analysis of *BnPYL* genes is sharing the similar motifs in each subfamily (Figure 4). However, *BnPYL* genes of the same group have similar functions, although we do not know the functions of these groups.

### 4.2. Expression Levels of BnPYLs in Various Tissues

*PYL* gene expression patterns in different tissues have been reported for many plants. In soybean, most *PYLs* are expressed at relatively higher levels in seeds than those in other soybean tissues [16]. In rubber tree, transcripts of *PYLs* are highly abundant in latex [19]. *PYLs* in *B. rapa* are expressed at a higher level in the callus than those in other tissues [20]. We analyzed the *PYL* gene expression patterns in the various tissues of oilseeds and found that *BnPYLs* show very high expression levels in seeds during germination stage (Figure 6). These results suggest that *PYLs* play important roles in regulating seed germination in *B. napus*, and that ABA signaling also participates in the regulation of seed germination in *B. napus*. The expression levels of 14 *BnPYLs* (*BnPYL2*, *BnPYL3*, *BnPYL4-1*, *BnPYL5-5*, *BnPYL10*, *BnPYL11*, *BnPYL12*, and *BnPYL13*) in all of the tissues were nearly zero in *B. napus*, suggesting that the functions of these genes are not required.

### 4.3. The Expression Patterns of BnPYLs under Abiotic Stresses

*B. napus* faces multiple abiotic stresses such as drought, high temperature and salinity, which seriously affect oilseed yields and seed quality. ABA has recently been reported to play crucial roles in responding to abiotic stresses, such as drought and salinity [44,45,46]. PYLs are involved in the initial step in ABA signal transduction. However, it is still unknown which *PYL* is the important ABA receptor in response to abiotic stress in *B. napus*. In our study, we selected 14 *BnPYLs* for qRT-PCR analysis and found that *PYR1-3*, *PYL1-2*, and *PYL7-2* were induced by heat, drought, and salinity stress, respectively (Figure 7), suggesting that these *PYLs* have multifunctional roles under various abiotic stresses. In addition, *PYL8-5* and *PYL8-6* were up-regulated in *B. napus* under drought stress, which is consistent with the results in cotton. In cotton, *GhPYL26*, which is homologous to the *PYL8* gene in *A. thaliana*, is overexpressed to enhance drought tolerance [21]. In a study by Zhao et al., *AtPYL9* overexpression promoted drought resistance in *A. thaliana* [25], whereas the expression levels of *BnPYL9-1* and *BnPYL9-2* were inhibited by drought and salinity stress. We thus speculated that this behavior may be explained by a negative-feedback regulatory mechanism: when a large quantity of ABA accumulates in the leaves under stress, *PYL* expression may be inhibited [47,48]. By contrast, the expression patterns of 14 *BnPYLs* under drought and salinity stress were similar. ABA is produced rapidly in response to stress under drought and salinity conditions and then plays an important role in the regulation of the stress response [49]. These findings and our data suggest that the *PYL* response mechanisms under drought and salinity stress may be similar. In conclusion, the results of the stress response experiments, combined with the analysis of the stress-responsive cis-elements in *BnPYL* promoters, suggest that some *BnPYLs* respond to drought, high temperature, and salinity treatments. These *PYLs* may potentially be utilized for improving the tolerance of *B. napus* to abiotic stresses.

## Figures and Tables

**Table 1:** *PYL* gene family information in *Brassica napus*.

Gene ID	Gene Name	Position (bp)	Gene Length (bp)	CDS Length (bp)	Exon	Peptide Residues	MW (kDa)	pI
*BnaCnng65400D*	*BnPYR1-1*	65121492-65122292	801	576	1	191	21.69	6.39
*BnaC07g34880D*	*BnPYR1-2*	37423726-37424722	997	576	1	191	21.40	5.98
*BnaAnng35310D*	*BnPYR1-3*	40055219-40055779	561	561	1	186	21.11	6.39
*BnaA03g43410D*	*BnPYR1-4*	21808356-21809284	929	576	1	191	21.37	5.98
*BnaC07g19450D*	*BnPYL1-1*	26187392-26188931	1540	648	1	215	24.38	5.29
*BnaA06g40360D*	*BnPYL1-2*	200261-2003467	854	633	1	210	23.91	5.20
*BnaA09g40690D*	*BnPYL2-1*	28565054-28565746	693	567	1	188	20.83	5.49
*BnaC08g33170D*	*BnPYL2-2*	31754064-31754755	692	576	1	188	21.03	5.86
*BnaC02g21700D*	*BnPYL3-1*	18634088-18634705	618	618	1	205	22.91	8.87
*BnaC03g23260D*	*BnPYL3-2*	12925626-12926243	618	618	1	205	22.91	8.88
*BnaA02g16230D*	*BnPYL3-3*	9682751-9683368	618	618	1	205	22.88	8.91
*BnaAnng13200D*	*BnPYL4-1*	14172422-14173048	627	627	1	208	22.57	7.08
*BnaA04g21960D*	*BnPYL4-2*	16651226-16652092	867	615	1	204	21.99	6.22
*BnaA03g17720D*	*BnPYL4-3*	8353447-8354313	867	624	1	207	22.24	6.43
*BnaC04g07010D*	*BnPYL4-4*	5159788-5160411	624	624	1	207	22.42	7.08
*BnaC03g21240D*	*BnPYL4-5*	11473885-11474748	864	624	1	207	22.29	6.43
*BnaC04g56560D*	*BnPYL4-6*	4191879-4192790	912	615	1	204	21.94	6.04
*BnaA10g24990D*	*BnPYL5-1*	16198838-16199452	615	615	1	204	22.78	6.02
*BnaC09g49910D*	*BnPYL5-2*	48035079-48036202	1124	615	1	204	22.77	5.82
*BnaAnng40650D*	*BnPYL5-3*	46553930-46554584	655	612	1	203	22.71	5.80
*BnaC03g02130D*	*BnPYL5-4*	994403-995014	612	612	1	203	22.68	5.91
*BnaAnng01330D*	*BnPYL5-5*	811157-811750	594	594	1	197	22.02	5.91
*BnaA05g05420D*	*BnPYL6-1*	2798876-2799514	639	639	1	212	23.48	6.52
*BnaA03g19030D*	*BnPYL6-2*	9003640-9004337	698	639	1	212	23.52	6.56
*BnaA04g29300D*	*BnPYL6-3*	1303160-1304112	953	618	1	205	22.77	6.09
*BnaC03g22610D*	*BnPYL6-4*	12499410-12500051	642	642	1	213	23.71	6.38
*BnaC04g47050D*	*BnPYL6-5*	46155948-46156559	612	612	1	203	22.50	6.10
*BnaC04g04830D*	*BnPYL6-6*	3526371-3527009	639	639	1	212	23.50	6.66
*BnaC03g31730D*	*BnPYL7-1*	19523342-19524503	1162	582	1	193	21.69	6.05
*BnaA03g26790D*	*BnPYL7-2*	13185542-13186722	1181	582	1	193	21.80	6.24
*BnaC02g14540D*	*BnPYL8-1*	10060041-10061474	1434	567	3	188	21.32	6.71
*BnaA03g12450D*	*BnPYL8-2*	5667935-5670048	2114	552	4	183	20.67	6.24
*BnaC03g15210D*	*BnPYL8-3*	7539236-7541464	2229	555	3	184	20.80	6.24
*BnaA02g10420D*	*BnPYL8-4*	5349136-5350394	1259	567	3	188	21.26	6.24
*BnaA10g06520D*	*BnPYL8-5*	4967767-4969159	1393	555	3	184	21.03	6.07
*BnaCnng37890D*	*BnPYL8-6*	36425463-36426990	1528	555	3	184	20.93	6.07
*BnaC05g00620D*	*BnPYL9-1*	331808-333031	1224	564	3	187	21.03	5.98
*BnaC05g17260D*	*BnPYL9-2*	10921616-10922842	1227	561	3	186	21.02	5.98
*BnaA10g00540D*	*BnPYL9-3*	270030-271246	1217	567	3	188	21.20	6.06
*BnaA01g16740D*	*BnPYL10-1*	8730925-8731696	772	558	3	185	20.78	5.61
*BnaCnng68710D*	*BnPYL10-2*	68363157-68363922	766	558	3	185	20.73	5.71
*BnaA06g40220D*	*BnPYL11*	1928871-1929359	489	489	1	162	17.82	5.40
*BnaCnng60010D*	*BnPYL12*	59845750-59846238	489	489	1	162	17.83	5.40
*BnaC01g11020D*	*BnPYL13-1*	6873982-6874482	501	501	1	166	18.41	5.12
*BnaA01g09460D*	*BnPYL13-2*	4636999-4637499	501	501	1	166	18.40	5.26
*BnaC07g48850D*	*BnPYL13-3*	1477983-1478483	501	501	1	166	18.41	5.26

MW: molecular weight; pI: isoelectric point.

## Supplementary Materials

The following are available online at http://www.mdpi.com/2073-4425/9/3/156/s1. Figure S1: The three highly conserved sequence logos in BnPYLs. The logo representations were generated using Weblogo. The sequence logos are the motifs which found to be conserved in 46 *BnPYL* proteins based on full-length alignments. The letters A, B, C denote motifs 1, 2 and 3, respectively. The *x*-axis represents the conserved protein sequences. The bit score shows the information content for each position in the *BnPYL* protein sequence. Asterisks in this figure are related to receptor activation. Table S1: Primers for qRT-PCR analysis of selected *BnPYL* genes; Table S2: Number of elements responsive to stresses and hormones in the promoter regions of *BnPYL* genes; Table S3: Expression levels of *BnPYL* genes in different tissues and organs of *B. napus* at different growth periods. The values represent the FPKM values; Table S4: The significantly enriched GO terms of 46 *BnPYLs*; Table S5: The expression levels of 14 *PYL* genes under drought, salinity and heat abiotic stresses in *B. napus*.
