# Supplementary figures and images for: Genome-Wide Analysis of the PYL Gene Family and Identification of PYL Genes That Respond to Abiotic Stress in Brassica napus
Source: Genes (Basel). 2018 Mar 12;9(3):156. doi: 10.3390/genes9030156 (PMC5867877; doi:10.3390/genes9030156)

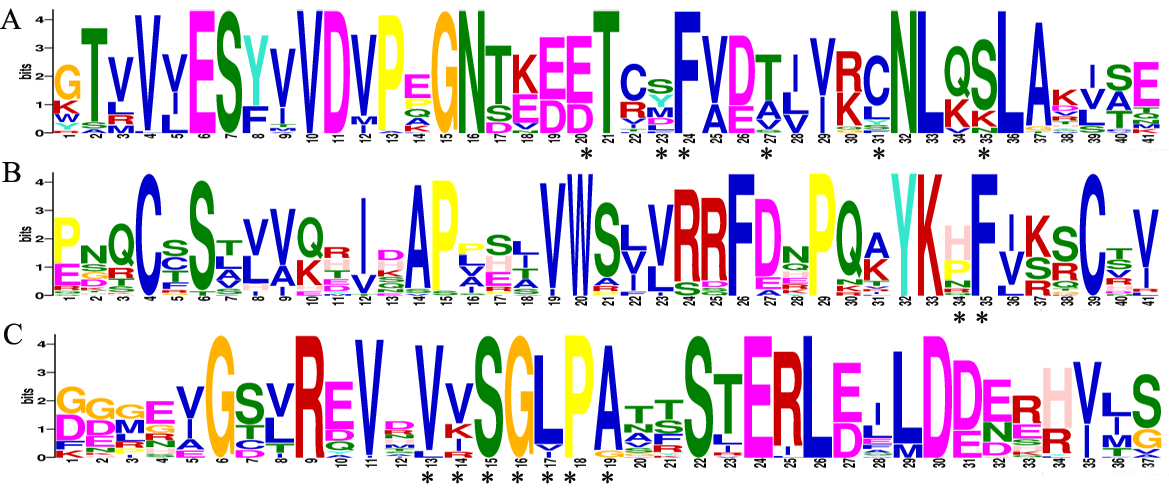

Supplement: Supplementary file 1 [file genes-09-00156-s001.zip › Supplementary File(s)/Figure S1.tif]
